# Ophthalmology: New Diagnostic and Treatment Approaches (Second Edition)

**DOI:** 10.3390/medicina62101961

**Published:** 2026-10-11

**Authors:** Stephen G. Schwartz, Andrzej Grzybowski, Victor M. Villegas, Christopher T. Leffler, Krishna S. Kishor

**Affiliations:** 1 Department of Ophthalmology, Bascom Palmer Eye Institute, University of Miami Miller School of Medicine, Naples, FL 34103, USA; 2 Foundation for Ophthalmology Development, 60-836 Poznan, Poland; ae.grzybowski@gmail.com; 3 Department of Ophthalmology, University of Puerto Rico School of Medicine, San Juan, PR 00936, USA; victor.villegas@upr.edu; 4 Department of Ophthalmology, Virginia Commonwealth University School of Medicine, Richmond, VA 23298, USA; chrislefflermd@gmail.com; 5 Department of Ophthalmology, Bascom Palmer Eye Institute, University of Miami Miller School of Medicine, Palm Beach Gardens, FL 33418, USA; kkishor@med.miami.edu

## Introduction

Many advances have been achieved in the diagnosis and treatment of eye diseases. The first edition of this Special Issue was completed in 2025, so a second edition was created to continue these efforts. As with the previous edition, the aims of this Special Issue are to publish new evidence and review the current state-of-the-art developments in the diagnosis and treatment of vision-threatening diseases. This edition features six original contributions, including four research articles and two review articles. These manuscripts cover advances in cataract surgery, age-related macular degeneration (AMD), myopic choroidal neovascularization (CNV), glaucoma, and dry eye disease.

Nowosielska and Rotuski studied the Technis Eyhance DIB00 posterior chamber intraocular lens (contribution 1). They retrospectively analyzed 214 eyes from 107 patients treated with simultaneous bilateral crystalline lens removal with implantation of these lenses and followed for one year. The uncorrected visual acuity (UCVA) and best-corrected visual acuity (BCVA) were UCVA 0.05/BCVA 0.02 LogMAR at distance; UCVA 0.03/BCVA −0.036 LogMAR at 70 cm; and UCVA 0.76/BCVA 0.18 LogMAR at 30 cm. No patients reported halos, glare, or diminished contrast sensitivity. The average patient satisfaction score was 8.54 out of 10 points.

Jovanovic et al. investigated the appearance of hyperreflective foci (HF) on optical coherence tomography in patients with neovascular AMD (contribution 2). They retrospectively studied 84 treatment-naïve eyes treated with intravitreal bevacizumab using a treat-and-extend protocol for six months. Intraretinal and subretinal HF and hyper-reflective material boundary remodeling were evaluated by two masked graders. The investigators reported that the intraretinal HF burden independently predicted visual outcomes in these patients.

Chang et al. examined outcomes after treatment with faricimab in patients with neovascular AMD (contribution 3). They retrospectively studied 76 eyes with treatment-naïve disease and 44 eyes with treatment-refractory disease treated with faricimab for up to three months. After one injection of faricimab, both treatment-naïve and treatment-resistant eyes showed significant reductions in central foveal thickness (CFT) but no significant improvements in BCVA. After three injections of faricimab in treatment-naïve eyes, BCVA and CFT significantly improved compared with baseline. 

Chang et al. evaluated treatment outcomes using faricimab in patients with myopic CNV (contribution 4). They retrospectively analyzed 27 eyes from 24 patients with myopic CNV (both treatment-naïve and previously treated with other agents) with a single injection of faricimab and evaluated at one month. After treatment, both the mean BCVA and mean central retinal thickness significantly improved compared with baseline. 

Rusciano et al. reviewed the current consensus on how abnormal cell motility contributes to the pathogenesis of glaucoma (contribution 5). Abnormal cell motility in the trabecular meshwork and Schlemm’s canal—regulated by Rho GTPase and ROCK signaling—contributes to impaired aqueous humor outflow and increased intraocular pressure. Abnormal cell motility of the optic nerve head glial cells—activated by mechanical stress and by TGF-β_2_ and other cytokines—contributes to fibrosis and neurodegeneration. 

Zeppieri et al. reviewed the effects of diabetes mellitus on dry eye disease (contribution 6). Hyperglycemia affects the ocular surface through a variety of mechanisms, including impaired lacrimal gland function, altered lipid secretions from Meibomian glands, and reduction in corneal nerve density, which contributes to neurotrophic keratitis. Dry eye disease may be a leading indicator of systemic disease in patients with diabetes. 

We think that these six contributions improve our current understanding of ophthalmology and may stimulate further beneficial research.

